# Electronic Structure and Phase Transition in Ferroelectic Sn_2_P_2_S_6_ Crystal

**DOI:** 10.3390/ijms131114356

**Published:** 2012-11-06

**Authors:** Konstantin Glukhov, Kristina Fedyo, Juras Banys, Yulian Vysochanskii

**Affiliations:** 1 Institute for Solid State Physics and Chemistry, Uzhgorod National University, Voloshyn Street 54, Uzhgorod 88000, Ukraine; E-Mails: k.fedyo@gmail.com (K.F.); vysochanskii@gmail.com (Y.V.); 2 Faculty of Physics, Vilnius University, LT-10222 Vilnius, Lithuania; E-Mail: juras.banys@ff.vu.lt

**Keywords:** ferroelectricity, phase transition, chemical bonding

## Abstract

An analysis of the P_2_S_6_ cluster electronic structure and its comparison with the crystal valence band in the paraelectric and ferroelectric phases has been done by first-principles calculations for Sn_2_P_2_S_6_ ferroelectrics. The origin of ferroelectricity has been outlined. It was established that the spontaneous polarization follows from the stereochemical activity of the electron lone pair of tin cations, which is determined by hybridization with P_2_S_6_ molecular orbitals. The chemical bonds covalence increase and rearrangement are related to the valence band changes at transition from the paraelectric phase to the ferroelectric phase.

## 1. Introduction

For such perovskite ferroelectrics as BaTiO_3_, the main origin of spontaneous polarization is commonly related to the hybridization interaction between the transition-metal and oxygen ions [1]. Another mechanism involves cations with “lone pair” electrons which have a formal *ns*^2^ valence electron configuration [2]. In the same manner as for the *d*^0^ transition-metal ions, these *p*^0^ ions (as example Pb^2+^ for PbTiO_3_, or Bi^3+^ for BiMnO_3_) contain some *p*-charge density which contribute to the displacive distortions. If the lowering of energy associated with the hybridization interaction is larger than the interionic repulsion opposing the ion shift, then a ferroelectric distortion appears. This “stereochemical activity of the lone pair” is the driving force for off-center distortion in ferroelectrics. Both named origins of ferroelectricity (the first *d*^0^-“ness” and the second “lone pair” activity) are familiar to the second-order Jahn–Teller (SOJT) effect [3,4]. This effect is determined by a balance of positive and negative contributions to the total energy. The first one describes short range repulsive forces and is related to the rigid ion (with frozen electronic configuration) shifts from original high symmetry positions. Such a term is small for the cases of “closed-shell” *d*^0^ or *p*^0^ cations. The second, negative, contribution describes the relaxation of electronic configuration in response to the ion displacements through covalent bonds formation. This term favors the ferroelectric distortion. For a full picture, the geometrical (or hybrid improper) mechanism, which is related to the rotational modes that trigger instability of polar mode [5,6], could also be considered in studying the ferroelectricity nature.

**Figure 1:**
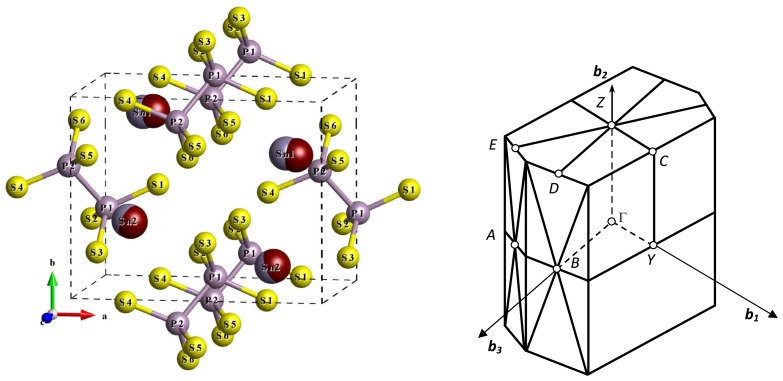
The crystal structure of Sn_2_P_2_S_6_ ferroelectric phase [20]. The tin atoms positions in the paraelectric phase [14] are shown in red. The shape of the Brillouin zone with denoted symmetrical points illustrates the primitive monoclinic lattice.

The cubic crystal lattices of ABO_3_ compounds are built by covalent bonds A–O and B–O with considerable contribution of ionicity for former ones. Naturally more complex bounding evolution could be supposed at the ferroelectric phase transition in ion-covalent crystal Sn_2_P_2_S_6_ with monoclinic lattice. For this compound the Sn^2+^ cations and the (P_2_S_6_)^4−^ anion clusters are joined by mostly ionic Sn–S bonds at covalent P–S and P–P bonding. Sn_2_P_2_S_6_ uniaxial ferroelectric undergoes the second order phase transition at *T*_0_*≈* 337 K (*P*2_1_*/n → Pn*, two formula units in the elementary cell for both phases (Figure 1)) in a crossover displacive-order/disorder region [7]. For the paraelectric phase, the optic lattice vibrations at the Brillouin zone center are distributed between irreducible presentations of 2*/m* point group in the following manner: Γ*_opt_* = 15*A_g_* + 15*B_g_* + 14*A_u_* + 13*B_u_*[7]. Ferroelectric instability in Sn_2_P_2_S_6_ crystal is a result of non-linear *A_g_B_u_*^2^ coupling of the soft polar *B_u_* and fully symmetrical *A_g_* optic modes, leading to three-well potential [8]. Here the opposite picture appears in comparison with perovskites where the only lattice mode could determine dynamical instability related to the ferroelectric phase transition [8]. In general, all the 13 *B_u_* and 15 *A_g_* optic modes were accounted in the frozen phonons approximation for construction of effective Hamiltonian for Sn_2_P_2_S_6_ crystal what was applied for the Monte-Carlo (MC) simulation of the ferroelectric phase transition and their behavior under hydrostatic pressure [9]. A system with a three-well potential was early considered by Lines [10,11] with application for the LiNbO_3_ and LiTaO_3_ crystals. Such a system can be generally described by two order parameters (related to dipole and quadruple moments), and as a result, a variety of stable, metastable and unstable states can be realized on a phase diagram [12,13].

The strong anharmonicity of the Sn_2_P_2_S_6_ crystal lattice is obviously joined with effective electron-phonon interaction, which appears as a stereochemical activity of the tin cations electron lone pair 5*s*^2^, and in fact, it is a reflection of the SOJT effect. The possible leading role of the cations’ stereoactivity for Sn_2_P_2_S_6_ crystals was noted early at X-ray structure investigations of their paraelectric phase in comparison with structure data for the ferroelectric phase [14]. Structural evidences of the tin cation stereoactivity was also analyzed in details by structure refinement of the paraelectric and the ferroelectric phases for Sn_2_P_2_Se_6_ selenide analog [15]. Through the Mössbauer effect investigations for the ^119^Sn nucleus [16] and the NMR spectroscopy for isotopes ^31^P and ^119^Sn [17,18], the important changes of chemical bonding at the ferroelectric phase transition in Sn_2_P_2_S_6_ were found. The X-ray photoelectron spectroscopy confirms growth of the chemical bonds covalence in the ferroelectric phase [19].

Sn_2_P_2_S_6_ crystals are ferroelectric semiconductors with promising photorefractive [21], photovoltaic [22], electrooptic [23] and piezoelectric [24] characteristics. Their ferroelectric properties are effectively influenced by the state of the electronic subsystem [25]. The influence of the sulfur and tin vacancies on semiconductive and optic properties of Sn_2_P_2_S_6_ crystals has recently been studied [26]. These data motivate the electronic structure investigation for Sn_2_P_2_S_6_ crystals in the paraelectric and the ferroelectric phases.

The first-principles calculations in LDA approach of Density Functional Theory (DFT) for Sn_2_P_2_S_6_ ferroelectric phase were carried out by several groups [27–29]. Grigas *et al.*[19,30] calculated the electronic structure of both paraelectric and ferroelectric phases of Sn_2_P_2_S_6_ using the cluster approach. For the Sn_2_P_2_Se_6_ selenide analog, the electronic structure have been investigated [31] by first-principles calculations only for the paraelectric phase. The electronic structure and phonon spectra pressure dependence for the acentric layered rhombohedral crystal SnP_2_S_6_ were investigated theoretically by the LDA approach [32]. For this compound, the tin cations are almost fully ionized (Sn^4+^ charge state), which excludes the possibility of stereochemical activity of their 5*s*^2^ electron lone pair. The electronic structure of high-charged (P_2_S_6_)^4−^ and (P_2_Se_6_)^4−^ anion clusters was discussed in papers [29,31,32] at analysis of Sn_2_P_2_S_6_, Sn_2_P_2_Se_6_ and SnP_2_S_6_ electron energy spectra. Analysis of P_2_S_6_ cluster chemical bounding was also done using the Hartree–Fock approach [33]. The anion arrangements have been inves- tigated experimentally and theoretically in different approximation for the layered crystals like M_2_P_2_S_6_ (M = Fe, Ni, Mn, . . . ) [34–38]. For the CuInP_2_Se_6_ layered compound with two differently charged cations, the SOJT effect was established as an origin of the ferroelectric ordering in Cu [39].

In this paper, the first-principles calculations in LDA approach of DFT for electronic structure of Sn_2_P_2_S_6_ crystal in the paraelectric and the ferroelectric phases were used for analysis of chemical bond transformation at the spontaneous polarization appearance and for establishing the ferroelectric state origin in the phosphorus-containing chalcogenides. As a background of the investigations, the free P_2_S_6_ structure group (atomic cluster) electron spectra and the peculiarities of their molecular orbitals were considered. The electron structure and chemical bonding nature in the paraelectric phase will also be analyzed. The stereochemical activity of the electron lone pair of tin cations is examined in detail. The growth of covalence and ion recharging are related to the spontaneous polarization.

Generally it was proposed that five steps could be divided hierarchically into a complex picture of electron-phonon interaction for the Sn_2_P_2_S_6_ ferroelectrics. At *first*, fully symmetrical *A_g_* modes spread the electronic charge from anionic complexes P_2_S_6_ and recharge cations Sn. This one induces hybridization of tin atomic orbitals with the anion’s molecular orbital, which appears as the cation’s stereochemical activity and could be considered as the *second* step. Subsequently, the *third* step is the weakening of the short-range repulsion between cations of tin and phosphorus, because their charges were lowered, in the presence of significant Coulomb repulsion by the inversion center nearest tin cations. Both the second and the third stages represent nonlinear interaction A_g_B_u_^2^ and determine the appearance of the structure dipoles related to the polar B_u_ modes. The *fourth* step is the dipole–dipole interaction which correlates orientation of local dipoles and defines the appearance of the spontaneous polarization. The *fifth* step will be included when all low symmetry modes participate as a result of permitted nonlinear A_u_B_g_B_u_ relation in the structure changes at the phase transition. The listed contributions into electron-phonon interaction will be investigated subsequently in the following parts of this paper. Finally, the influence of substituting tin with lead and substituting sulfur with selenium on the crystals properties is discussed.

## 2. Method of Calculations

The calculations of the band structure of both phases of Sn_2_P_2_S_6_ crystal as well as energy levels of P_2_S_6_ molecule has been performed by means of the package program ABINIT [40] (total and projected densities of states were calculated using SIESTA [41] software) in the framework of the DFT, using the local density approximation for representing the exchange–correlation interaction. A basis set of 28,000 plane waves restricted by the kinetic energy *E_cut_* = 25 Hartree has been used. The tin, sulphur and phosphorus atoms had the following electron configurations: Sn: [Kr] 5*s*^2^5*p*^2^; S: [Ne] 3*s*^2^3*p*^4^; and P: [Ne] 3*s*^2^3*p*^3^, respectively. The “frozen” core electron configurations for each atom is shown in brackets. The first-principles pseudopotentials in the Hartwigsen–Goedecker–Hutter scheme [42] have been applied and the integration over irreducible part of the Brillouin zone has been done by means of the tetrahedron method using the 4 *×* 4 *×* 4 Monkhorst–Pack mesh [43] of **k**-points. The chosen parameters were sufficient for a good convergence in the calculations. Prior to commencing the calculation of physical properties of Sn_2_P_2_S_6_ crystal, we carried out the structural optimization, which minimized total energy of the system simultaneously with the forces [44] acting on atoms. The spin-orbit interaction was not taken into account in our calculation.

The parameters of Sn_2_P_2_S_6_ crystal obtained after structural relaxation can be compared with experimental data, presented in [14,20]. The comparison of experimental and calculated relaxed values of lattice constants demonstrate the difference of about 3% in the order of magnitude. Also let us note that the necessity of ABINIT code usage was caused by our intention to perform group theory analysis of the wave functions, and the plane wave bases used in this code perfectly fit for this purpose.

## 3. Electronic Structure of P_2_S_6_ Cluster

The molecular orbitals of P_2_S_6_ cluster create covalent P–S and P–P bonds. Their hybridization with tin atomic orbitals determines the electronic structure of Sn_2_P_2_S_6_ crystal. The electronic energy spectra of this material could be analyzed by the calculation of a free P_2_S_6_ cluster electronic structure followed by accounting their molecular orbital hybridization with the atomic orbitals of tin.

**Figure 2:**
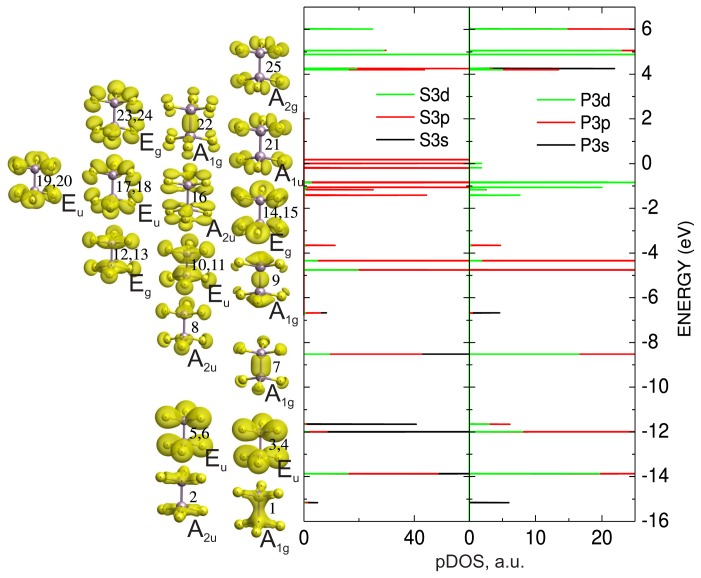
The partial densities of states and space distribution of electron density for the molecular orbitals of P_2_S_6_ cluster in free state.

The calculated energy spectrum and partial densities of electron states for *s*, *p* and *d* orbitals of phosphorus and sulfur atoms of a free P_2_S_6_ molecule illustrate the formation of different molecular orbitals upon the creation of covalent P–S and P–P bonds. The spatial electron density distribution for related energy levels reflects peculiarities of these bonds. It is seen that for clusters, the energy level near −15 eV is mostly determined by hybridization of phosphorus *s* orbitals. Here some contributions of sulfur *s* and *p* orbitals have been also found. The hybridization of these atomic orbitals (Scheme 1 in Figure 2) creates bonding P–P and P–S molecular orbitals. The level near −14 eV is determined by antibonding combination of two phosphorus *s* orbitals and by bonding hybridization of *s* orbitals (2) of phosphorus and sulfur atoms.

The group of levels near −12 eV is mostly formed by sulfur *s* orbitals. These levels are related to the P–S bonding and P–P antibonding molecular orbitals (3,4,5,6). The levels near −6.5 eV and −8.5 eV appear as a replica of the doublet of the levels near −15 eV and −14 eV due to antibonding hybridization of phosphorus and sulfur atoms *s* orbitals. The molecular orbitals for levels near −8.5 eV are P–P bonding and P–S antibonding (7), the orbitals with energy near −6.5 eV are antibonding for all P–S and P–P bonds (8). In intervals between −3.5 and −4.7 eV, the energy levels are created by bonding hybridization of phosphorus and sulfur *p* orbitals. These orbitals (9, 10, 11, 12, 13) are bonding for P–P and P–S bonds. In region from 0 to −1.5 eV, the energy levels of the cluster are also formed by *p* orbitals of phosphorus and sulfur atoms. They are hybridized in P–P bonding and P–S antibonding molecular orbitals (14–23). Here the contribution from phosphorus *d* orbitals is also presented.

In the presented work we perform the group theory analysis of the states of the P_2_S_6_ molecule. It results in a set of irreducible representations that describes the symmetry of its states. They are the following:

$$A_{1g},A_{2u},E_{u},E_{u},A_{1g},A_{2u},A_{1g},E_{u},E_{g},E_{g},A_{2u},E_{u},E_{u},A_{1u},A_{1g},E_{g}↓A_{2g},A_{1g},A_{2u},\ldots$$

Occupied and unoccupied states are separated by an arrow. If we use the well-known criteria of the Jahn–Teller effect occurrence and find the direct square of its most occupied state, we obtain the following:

$$E_{u}\times E_{u}=A_{1g}+A_{2g}+E_{u}$$

As far as the right hand side of the above expansion contains the asymmetric representations, the corresponding normal modes will break the *D*_3_*_d_* symmetry of the P_2_S_6_ molecule (Jahn–Teller effect). Also, it is worth noting that the 23rd and 24th energy levels (*E_g_*) of the P_2_S_6_ molecule are double degenerated due to high symmetry but only half occupied. Such peculiarity can increase instability of this complex by means of the Jahn–Teller-like mechanism.

From a detailed analysis of the electron energy spectrum, the important information about creation and character of the chemical bonds in the P_2_S_6_ cluster could be found (Figure 2). The bond P–P is determined by *σ* hybridization of phosphorus *s* orbitals (the level near −15 eV (1)) and by their replica near −8.5 eV (7). In P–P bond, the contribution from *π* hybridization of *p_x_* and *p_y_* orbitals of phosphorus, which are oriented normally to the bond direction, is also presented. The levels of these orbitals (9) are placed near −4 eV. However, the essential contribution into energy of the P–P bond adds the *σ* hybridization of phosphorus *p_z_* orbitals that are oriented along the bond. The bonding combination *σ* (*p_z_* + *p_z_*) (22) has been filled by electrons with the energy level in the range of 0 to −1.5 eV. Also the nonbonding combination σ^*^ (*p_z_* – *p_z_*), which is related to the empty energy level, has been found in this energy region. Some contribution to the bond between the PS_3_ structure pyramids of P_2_S_6_ cluster also came from hybridization of *p* orbitals of sulfur atoms that belong to different pyramids.

By *σ* hybridization of phosphorus and sulfur *s* orbitals, the P–S bonds are created. Also the hybridization of *p* orbitals of phosphorus and sulfur atoms is observed (Figure 2). Such hybridization has obviously both *σ* and *π* character. Thus the next scheme for the appearance of molecular orbitals that form P–S bonds in PS_3_ structural pyramid could be proposed. The phosphorus atom realizes *sp*^2^ hybridization from which three symmetrically oriented bonds involving sulfur *p* orbitals appear. Two electrons from surrounding cations in a crystal lattice and one *s* electron of phosphorus atom (excited on *d* orbital) fill the covalent P–S bonds.

In Sn_2_P_2_S_6_ crystal, all energy levels for P_2_S_6_ clusters are occupied, and in the ionic bonding approach, the charge states S^2−^ for sulfur ions and P^4+^ for phosphorus ions are expected. However, as it follows from calculations for P_2_S_6_ molecular orbitals, enough high charge density is found at phosphorus atoms. By this matter, not high positive charge (drastically smaller than +4) is expected for the phosphorus ions. In addition, the calculations provide evidence about high electronic density at the middle of P–P bond.

## 4. Electronic Structure of Sn_2_P_2_S_6_ Crystal

When building the Sn_2_P_2_S_6_ crystal structure with two formula units in the elementary cell (Figure 1), the quantity of energy levels of P_2_S_6_ clusters is doubled with their energies splitting due to the intercluster interaction. Also, the energy levels of 5*s*^2^ orbitals of four Sn^2+^ cations are added to the structure of the crystal valence band (VB). The cation’s 5*p*^2^ orbitals participate in the formation of the conduction band of crystal. The scheme of electron orbital hybridization in the crystal could be presented as additive combination of the above described scheme of P_2_S_6_ cluster orbitals creation and the scheme of hybridization of these molecular orbitals with tin atomic orbitals. Generally, for the crystal, four tin atoms, four phosphorus atoms and twelve sulfur atoms in the elementary cell have 108 valence electrons that are placed at 54 energy levels in the VB.

In our work, we also perform the group theory analysis of the energy states of Sn_2_P_2_S_6_ crystal in set of high symmetry points of its Brillouin zone. As it was mentioned above, the crystal lattice of Sn_2_P_2_S_6_ compound in the paraelectic phase has the symmetry of the P2_1_*/*n space group. Corresponding symmetry operations can be written down in the following form:

$$\left\{E(x,y,z)|0\};\;\;\;\{{\tilde{C}}_{2}(-x,y,-z)|\vec{\alpha }\};\;\;\;\{I(-x,-y,-z)|0\};\;\;\;\{{\tilde{\sigma }}_{h}(x,-y,z)|\vec{\alpha }\right\}$$

where nontrivial translation *α⃗* = (*α⃗*_1_ +*α⃗*_2_ +*α⃗*_3_)*/*2. This group has one-dimensional irreducible representations (irreps) in points Γ(0*,* 0*,* 0) and $A(-\frac{1}{2},0,\frac{1}{2})$ (Table 1). Irreps in all other high symmetry points are two-dimensional. But in $D(0,\frac{1}{2},\frac{1}{2})$ and $C(\frac{1}{2},\frac{1}{2},0)$ points, this two-dimensionality is connected with time-reversal invariance and the corresponding little groups have two (even and odd) coupled irreps (Table 2). All other little groups (for points $B(0,0,\frac{1}{2}),Y(\frac{1}{2},0,0),Z(0,\frac{1}{2},0)$ and $E(-\frac{1}{2},\frac{1}{2},\frac{1}{2})$ have only one single two-dimensional irrep (Table 3). Our symmetry analysis of calculated wave functions results in the sets of the following irreps:

$$\begin{array}{c} \Gamma_{1},\Gamma_{3},\Gamma_{2},\Gamma_{4},\Gamma_{2},\Gamma_{4},\Gamma_{3},\Gamma_{2},\Gamma_{4},\Gamma_{3},\Gamma_{1},\Gamma_{1},\Gamma_{1},\Gamma_{3},\Gamma_{2},\Gamma_{4},\Gamma_{3},\Gamma_{4},\Gamma_{2},\Gamma_{3},\Gamma_{1},\Gamma_{4},\Gamma_{4},\Gamma_{2},\Gamma_{1},\\ \Gamma_{3},\Gamma_{2},\Gamma_{1},\Gamma_{1},\Gamma_{3},\Gamma_{1},\Gamma_{4},\Gamma_{1},\Gamma_{2},\Gamma_{4},\Gamma_{3},\Gamma_{2},\Gamma_{3},\Gamma_{1},\Gamma_{4},\Gamma_{2},\Gamma_{4},\Gamma_{2},\Gamma_{1},\Gamma_{3},\Gamma_{4},\Gamma_{3},\Gamma_{1},\Gamma_{3},\Gamma_{2},\\ \Gamma_{1},\Gamma_{4},\Gamma_{3},\Gamma_{2}↓\Gamma_{2},\Gamma_{3},\Gamma_{2},\Gamma_{4},\Gamma_{4},\Gamma_{2}\ldots \\ A_{2},A_{4},A_{1},A_{3},A_{3},A_{1},A_{3},A_{4},A_{2},A_{1},A_{2},A_{4},A_{2},A_{4},A_{1},A_{3},A_{4},A_{3},A_{2},A_{1},A_{1},A_{2},A_{3},A_{4},A_{3},\\ A_{1},A_{2},A_{4},A_{4},A_{2},A_{4},A_{1},A_{2},A_{3},A_{3},A_{1},A_{3},A_{4},A_{2},A_{1},A_{3},A_{1},A_{2},A_{4},A_{2},A_{4},A_{2},A_{1},A_{3},A_{4},\\ A_{2},A_{1},A_{3},A_{4}↓A_{4},A_{2},A_{1},A_{3},A_{3},A_{2}\ldots \\ \{D_{1}⊕D_{3}\},\{D_{2}⊕D_{4}\},\{D_{2}⊕D_{4}\},\{D_{2}⊕D_{4}\},\{D_{1}⊕D_{3}\},\{D_{1}⊕D_{3}\},\{D_{1}⊕D_{3}\},\{D_{2}⊕D_{4}\},\\ \{D_{1}⊕D_{3}\},\{D_{2}⊕D_{4}\},\{D_{2}⊕D_{4}\},\{D_{1}⊕D_{3}\},\{D_{2}⊕D_{4}\},\{D_{1}⊕D_{3}\},\{D_{1}⊕D_{3}\},\{D_{2}⊕D_{4}\},\\ \{D_{2}⊕D_{4}\},\{D_{1}⊕D_{3}\},\{D_{2}⊕D_{4}\},\{D_{1}⊕D_{3}\},\{D_{1}⊕D_{3}\},\{D_{2}⊕D_{4}\},\{D_{1}⊕D_{3}\},\{D_{2}⊕D_{4}\},\\ \{D_{2}⊕D_{4}\},\{D_{1}⊕D_{3}\},\{D_{1}⊕D_{3}\}↓\{D_{1}⊕D_{3}\},\{D_{2}⊕D_{4}\},\{D_{1}⊕D_{3}\}\ldots \\ \{C_{2}⊕C_{4}\},\{C_{1}⊕C_{3}\},\{C_{1}⊕C_{3}\},\{C_{1}⊕C_{3}\},\{C_{2}⊕C_{4}\},\{C_{2}⊕C_{4}\},\{C_{2}⊕C_{4}\},\{C_{1}⊕C_{3}\},\\ \{C_{2}⊕C_{4}\},\{C_{1}⊕C_{3}\},\{C_{2}⊕C_{4}\},\{C_{1}⊕C_{3}\},\{C_{1}⊕C_{3}\},\{C_{2}⊕C_{4}\},\{C_{2}⊕C_{4}\},\{C_{1}⊕C_{3}\},\\ \{C_{2}⊕C_{4}\},\{C_{1}⊕C_{3}\},\{C_{1}⊕C_{3}\},\{C_{2}⊕C_{4}\},\{C_{2}⊕C_{4}\},\{C_{1}⊕C_{3}\},\{C_{1}⊕C_{3}\},\{C_{2}⊕C_{4}\},\\ \{C_{2}⊕C_{4}\},\{C_{1}⊕C_{3}\},\{C_{2}⊕C_{4}\}↓\{C_{1}⊕C_{3}\},\{C_{2}⊕C_{4}\},\{C_{1}⊕C_{3}\}\ldots \end{array}$$

Occupied (valence band) and unoccupied (conduction band) states are separated by arrow. States coupled due to time-reversal invariance condition are placed in brackets.

**Figure 3:**
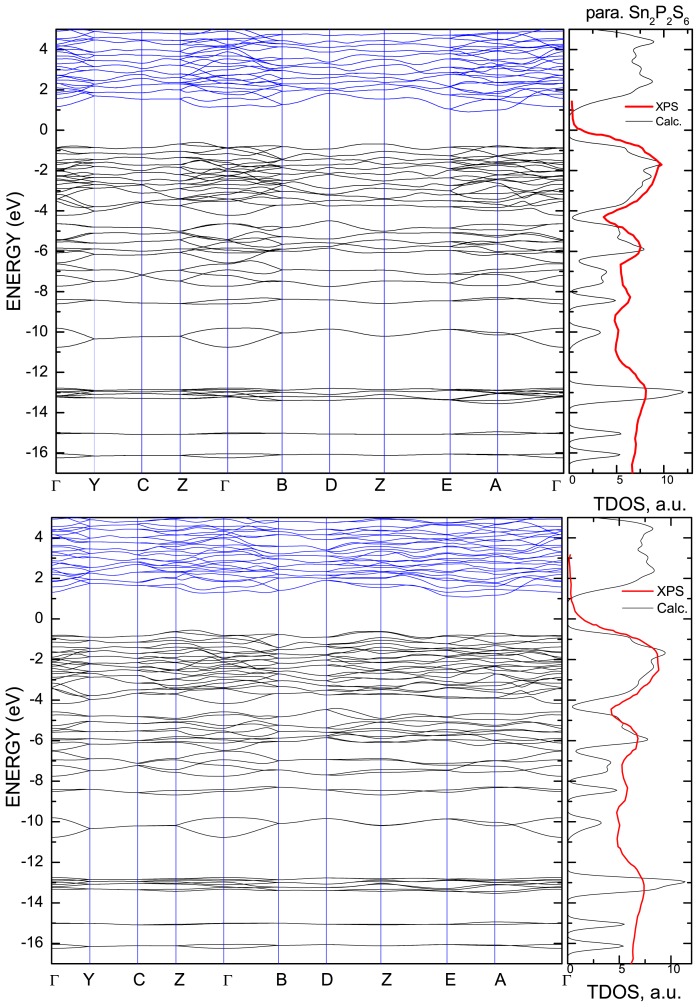
The electron energy spectrum of Sn_2_P_2_S_6_ crystal in the paraelectric (top) and the ferroelectric (bottom) phases. The calculated total density of states is compared with experimental XPS data [19].

As it can be easily seen from the band structure (Figure 3), all these states form complexes with the structure:

$$\begin{array}{c} \Gamma_{1}⊕\Gamma_{3}-A_{2}⊕A_{4}-\{D_{1}⊕D_{3}\}-\{C_{2}⊕C_{4}\};\\ \Gamma_{2}⊕\Gamma_{4}-A_{1}⊕A_{3}-\{D_{2}⊕D_{4}\}-\{C_{1}⊕C_{3}\};\\ \Gamma_{1}⊕\Gamma_{2}⊕\Gamma_{3}⊕\Gamma_{4}-A_{1}⊕A_{2}⊕A_{3}⊕A_{4}-\{D_{1}⊕D_{3}\}⊕\{D_{2}⊕D_{4}\}-\{C_{1}⊕C_{3}\}⊕\{C_{2}⊕C_{4}\}. \end{array}$$

By comparing these complexes with the representations induced by the irreps of site symmetry groups for all Wyckoff positions of the Sn_2_P_2_S_6_ crystal elementary cell, one can find the so-called *actual* Wyckoff positions [45]. In our case two first complexes originate from Wyckoff position $d(\frac{1}{2},0,0)$, which is the point in the middle of the P–P bond and the last one corresponds to general position *e*(*x, y, z*). Physically, the actuality of some positions means that the maxima of spatial charge distribution will be in the vicinity of these geometrical manifolds.

Analogous symmetry analysis was also performed for the ferroelectric phase of Sn_2_P_2_S_6_ crystal. The corresponding space group is *Pn*. It contains only two symmetry operations:

$$\left\{E(x,y,z)|0\};\;\;\;\{{\tilde{\sigma }}_{h}(x,-y,z)|\vec{\alpha }\right\}$$

Here nontrivial translation *α⃗* is the same as in the previous case.

Energy states in high symmetry points Γ(0*,* 0*,* 0) and $A(-\frac{1}{2},0,\frac{1}{2})$ remain one-dimensional. Due to symmetry lowering in points $Z(0,\frac{1}{2},0)$ and $E(-\frac{1}{2},\frac{1}{2},\frac{1}{2})$, energy states become non-degenerate (see Table 4). But in points $D(0,\frac{1}{2},\frac{1}{2}),C(\frac{1}{2},\frac{1}{2},0),B(0,0,\frac{1}{2})$ and $Y(\frac{1}{2},0,0)$, degeneration holds because of time-reversal invariance (Table 5).

The symmetry of calculated wave functions for the set of high symmetry points of the Brillouin zone has been written below.

$$\begin{array}{c} \Gamma_{1},\Gamma_{2},\Gamma_{2},\Gamma_{1},\Gamma_{2},\Gamma_{1},\Gamma_{2},\Gamma_{2},\Gamma_{1},\Gamma_{2},\Gamma_{1},\Gamma_{1},\Gamma_{1},\Gamma_{2},\Gamma_{2},\Gamma_{1},\Gamma_{2},\Gamma_{1},\Gamma_{2},\Gamma_{2},\Gamma_{1},\Gamma_{1},\Gamma_{1},\Gamma_{2},\Gamma_{1},\\ \Gamma_{2},\Gamma_{2},\Gamma_{1},\Gamma_{1},\Gamma_{2},\Gamma_{1},\Gamma_{1},\Gamma_{2},\Gamma_{1},\Gamma_{2},\Gamma_{1},\Gamma_{2},\Gamma_{2},\Gamma_{1},\Gamma_{1},\Gamma_{2},\Gamma_{2},\Gamma_{1},\Gamma_{1},\Gamma_{2},\Gamma_{1},\Gamma_{2},\Gamma_{1},\Gamma_{2},\Gamma_{2},\\ \Gamma_{1},\Gamma_{1},\Gamma_{2},\Gamma_{2}↓\Gamma_{2},\Gamma_{2},\Gamma_{2},\Gamma_{1},\Gamma_{1},\Gamma_{1}\ldots \\ A_{2},A_{2},A_{1},A_{1},A_{1},A_{1},A_{2},A_{1},A_{2},A_{1},A_{2},A_{2},A_{2},A_{2},A_{1},A_{1},A_{2},A_{1},A_{1},A_{2},A_{1},A_{2},A_{1},A_{2},A_{1},\\ A_{1},A_{2},A_{2},A_{2},A_{2},A_{1},A_{2},A_{1},A_{2},A_{1},A_{1},A_{1},A_{2},A_{2},A_{1},A_{1},A_{1},A_{2},A_{2},A_{2},A_{2},A_{2},A_{1},A_{1},A_{2},\\ A_{2},A_{1},A_{1},A_{2}↓A_{2},A_{2},A_{1},A_{1},A_{1},A_{2}\ldots \\ Z_{2},Z_{1},Z_{2},Z_{1},Z_{1,}Z_{2,}Z_{1,}Z_{2,}Z_{2,}Z_{1,}Z_{1,}Z_{2,}Z_{1,}Z_{2,}Z_{2,}Z_{1,}Z_{2,}Z_{1,}Z_{2,}Z_{1,}Z_{1,}Z_{2,}Z_{2,}Z_{1,}Z_{1,}\\ Z_{2},Z_{2},Z_{1},Z_{1},Z_{2,}Z_{2,}Z_{1,}Z_{1,}Z_{1,}Z_{2,}Z_{2,}Z_{1,}Z_{2,}Z_{2,}Z_{1,}Z_{2,}Z_{1,}Z_{1,}Z_{2,}Z_{1,}Z_{2,}Z_{2,}Z_{1,}Z_{2,}Z_{1,}\\ Z_{1},Z_{2},Z_{1},Z_{2}↓Z_{1,}Z_{2,}Z_{2,}Z_{1,}Z_{2,}Z_{1}\ldots \\ E_{2},E_{1},E_{1},E_{2},E_{1},E_{2},E_{2},E_{1},E_{1},E_{2},E_{2},E_{1},E_{2},E_{1},E_{1},E_{2},E_{1},E_{2},E_{1},E_{2},E_{1},E_{2},E_{2},E_{1},E_{2},\\ E_{1},E_{2},E_{1},E_{2},E_{1},E_{2},E_{1},E_{2},E_{1},E_{2},E_{1},E_{2},E_{1},E_{2},E_{1},E_{1},E_{2},E_{2},E_{1},E_{1},E_{2},E_{1},E_{2},E_{2},E_{1},\\ E_{1},E_{2},E_{2},E_{1}↓E_{1},E_{2},E_{1},E_{2},E_{1},E_{2}\ldots \end{array}$$

The states of the valence and conduction bands are separated by arrow.

It is interesting to note that the elementary cell of the ferroelectric phase Sn_2_P_2_S_6_ contains only a general Wyckoff position *a*(*x, y, z*). Therefore it is an actual one. Also it means that band representation must contain all irreps in one complex. But if we take a glance on the separated states with the lowest energy scale in point *A*, we can notice that the corresponding couple of wave functions has the symmetry of *A*_2_. To obtain the correct closed complex, we must combine four lower dispersion branches together. This symmetry-based peculiarity causes a strong coupling between the mentioned states, which originate from P–S and P–P bonds and can be essential for the stability of the (P_2_S_6_)^4−^ anion.

**Figure 4:**
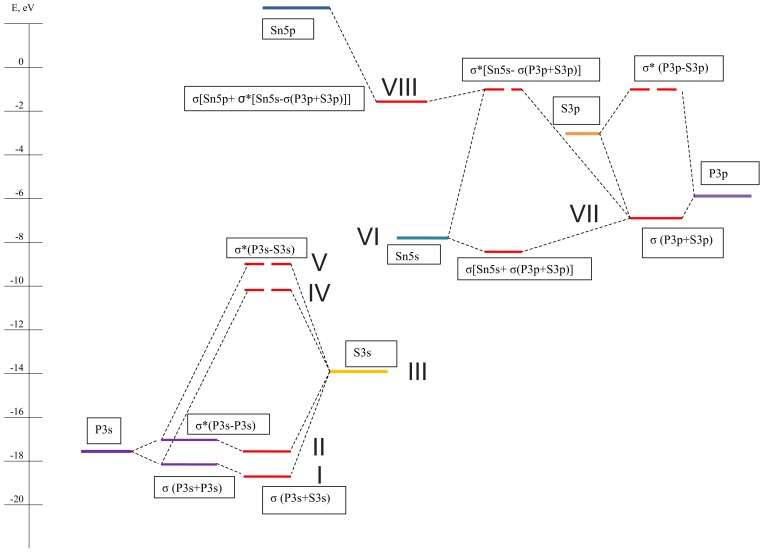
The hybridization scheme for electronic orbitals in Sn_2_P_2_S_6_ crystal.

It is known [46] that energy positions of the atomic orbitals of phosphorus, sulfur and tin are: P 3*p* = −8.35 eV, P 3*s* = −17.13 eV; S 3*p* = −10.28 eV, S 3*s* = −20.8 eV; Sn 5*p* = −4 eV, Sn 5*s* = −11 eV. The scheme of hybridization (Figure 4) could be proposed, which is in agreement with calculated energy spectra for free P_2_S_6_ cluster (Figure 2) and for Sn_2_P_2_S_6_ crystal (Figure 3). During the crystal structure formation, the energy of phosphorus valence orbitals remains almost unchanged while the bonding energy for sulfur valence orbitals decreases strongly (almost by 7 eV). This is in agreement with the raised electronic density on the sulfur anions and the enough high electronic density surrounding the phosphorus atoms.

**Figure 5:**
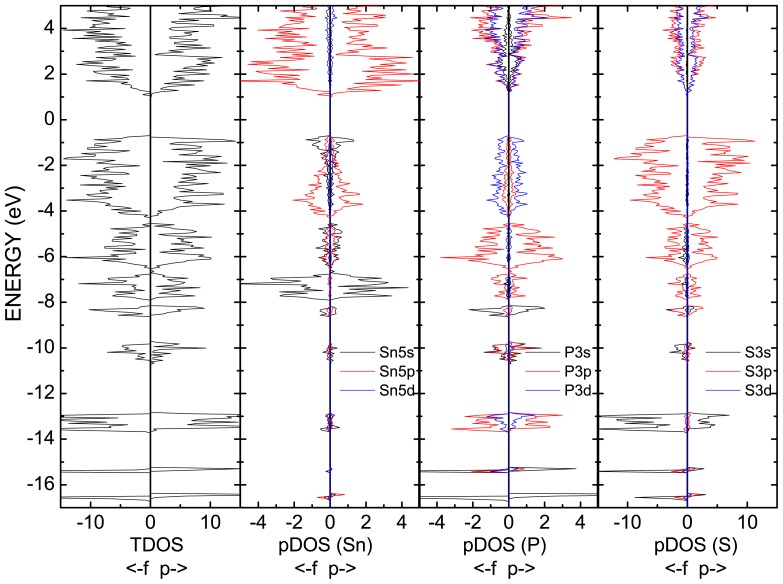
The total and partial electron densities of states for Sn_2_P_2_S_6_ crystal in the paraelectric (denoted by p) and ferroelectric (denoted by f) phases.

In accordance to the calculated energy spectra and densities of states of Sn_2_P_2_S_6_ crystal (Figures 3 and 5), its VB could be divided into eight subbands, which are labeled in the hybridization scheme of atomic and molecular electronic orbitals. Remember that for the energy spectrum of a free P_2_S_6_ cluster only seven subbands were found (Figure 2). For Sn_2_P_2_S_6_ crystal, the additional levels of tin atomic *s* orbitals are placed near −8 eV and they are related to the sixth subband of the VB.

By comparison of calculated partial densities of states for para- and ferrophases, one can notice slight energy downshift of all states and increased valence band width in ferrophase. This corresponds to the general increase in bonding covalence at phase transition.

**Figure 6:**
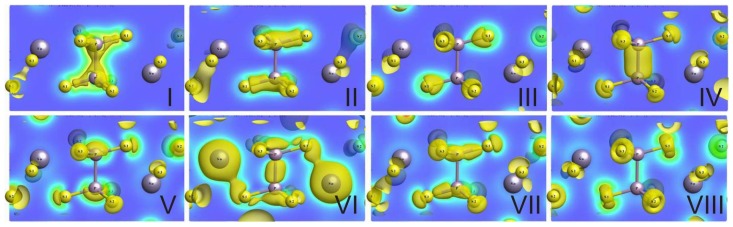
The spatial charge density distribution (in plane which contains S–P–P–S bonds) for orbitals in the valence subbands I–VIII for the paraelectric phase of Sn_2_P_2_S_6_ crystal.

The subband I contains two energy levels near −16.5 eV, for which a contribution of phosphorus *s* orbitals dominates (Figure 5). Here, a small contribution of sulfur *s* orbitals is also presented, and a minor appearance of sulfur *p* orbitals is observed. The contribution of tin atom valence orbitals in this subband is specific peculiarity of the crystal energy spectrum. Generally, the bonding orbitals of covalent P–P and P–S bonds are created in subband I (Figure 6).

The subband II includes the two energy levels near −15.5 eV. It is formed by the antibonding combination of the *s* orbitals of two phosphorus atoms and by the bonding hybridization of the *s* orbitals of phosphorus and sulfur atoms. For these levels, some contribution from phosphorus *d* orbitals is also observed.

The subband III in region from −13 eV to −13.8 eV has eight energy levels for which electron charge density is mostly localized at sulfur atoms (Figure 6). Here the contribution of phosphorus 3*p* and 4*d* orbitals is also presented. This subband is characterized by bonding hybridization for P–S and antibonding hybridization for P–P covalent bonds in the crystal structure.

The subbands IV and V near −10 eV and −8.5 eV (both of which containing two energy levels) are formed by *s* and *p* orbitals of phosphorus and sulfur atoms. They are the replica of the subbands II and I and originate from their hybridization with subband III. For subband IV, the charge is mostly localized at phosphorus atoms and it has a bonding character for the P–P bonds and an antibonding character for the P–S bonds. The subband V has antibonding character for both P–P and P–S bonds.

The subband VI with four energy levels in range from −8 up to −6.5 eV appears in the VB of Sn_2_P_2_S_6_ crystal as a result of hybridization of tin atomic electron orbitals with P_2_S_6_ clusters molecular orbitals. This subband is mainly formed by tin *s* orbitals and by *p* orbitals of phosphorus and sulfur (Figure 5). The charge of this subband’s hybridized orbitals is located between phosphorus atoms and around tin atoms and has Sn–S and P–P bonding character (Figure 6). The peculiarities of orbital hybridization for each of the four levels in this subband will be analyzed in details later, with the aim of explaining interatomic interactions that are related to the ferroelectric phase transition in Sn_2_P_2_S_6_ crystal.

The subband VII located in energy region between −6.5 and −4.5 eV contains ten energy levels. They are formed by phosphorus and sulfur *p* orbitals and also include small contribution of tin electronic orbitals. This subband has bonding character for P–S and P–P bonds and has antibonding character for the Sn–S bonds.

Subband VIII is situated near the top of crystal’s valence band and includes 24 energy levels in the energy range from −4.5 to −0.5 eV. The considered subband is mostly formed by lone pairs of sulfur *p* orbitals, with some participation of phosphorus *p* orbitals, and it has P–P bonding and P–S antibonding character. Here the hybridization of the *s* and *p* orbitals of tin atoms, which determines their stereoactivity (Figure 6), is also reproduced. The nature of the stereochemical activity of Sn^2+^ cations in the Sn_2_P_2_S_6_ crystal structure will be analyzed in details later on.

## 5. Hybridization of Tin Atomic Orbitals with Molecular Orbitals of P_2_S_6_ Clusters

**Figure 7:**
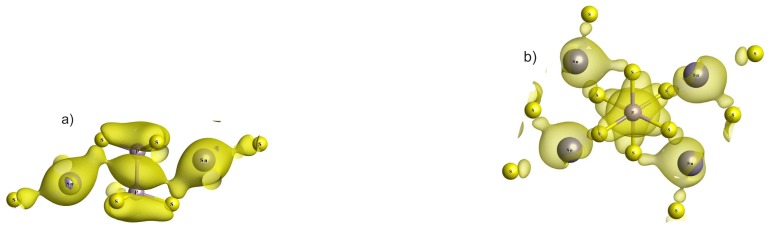
The electron density distribution (**a**) in plane parallel and (**b**) perpendicular to P–P bond for orbitals of energy level 20 in the valence subband VI for the paraelectric phase of Sn_2_P_2_S_6_ crystal.

As it was mentioned above, the valence subband VI includes four levels in the region from −8 to −6.5 eV, which are related to *s* orbitals of tin atoms (Figures 3–6). These are levels from 17 to 20, and their spatial electronic charge distribution illustrates bonding peculiarities of tin atomic orbitals with sulfur and phosphorus orbitals, which create the clusters P_2_S_6_. Level 17 is characterized by enough strong bonding of tin atoms with two nearest sulfur atoms, and levels 18 and 19 demonstrate strong bonding of tin atom with one of the nearest sulfur atoms. The spatial charge distribution of level 20 is of special interest—here the electron density is elongated from tin atom to the middle of P–P bond inside the P_2_S_6_ cluster (Figure 7a).

It can be easily seen that in the paraelectric phase, in addition to the –Sn–S–P–P–S–Sn– sequence of chemical bonds, the –Sn–P–P–Sn– series also exists. Such sequence of direct bonds of tin atoms with phosphorus atoms appears due to anisotropy of the spatial charge distribution for level 20 (Figure 7b). Named distribution has a form of layers that are oriented close to the plane (101). It is important that the directions of the shifts of tin atoms during transition into ferroelectric phase [14,20] are also rather close to the orientation of the mentioned plane.

**Figure 8:**
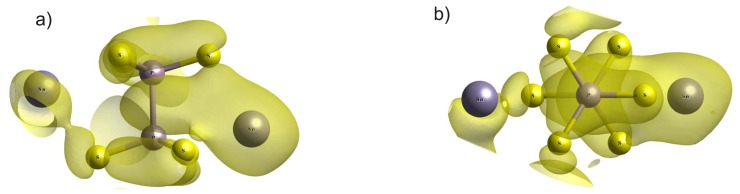
The electron charge distribution (**a**) in plane parallel and (**b**) perpendicular to the P–P bond that illustrates the hybridization of tin and phosphorus orbitals for energy level 20 in the valence subband VI of the Sn_2_P_2_S_6_ ferroelectric phase.

In the ferroelectric phase, the two pairs of nonequivalent tin atoms are presented in the crystal structure, which is clearly illustrated by the spatial distribution of the electron charge density for level 20 (Figure 8a). Near the mentioned P_2_S_6_ cluster, one of the tin atoms (denoted as Sn_2_) approaches the middle of the P–P bond. The electron charge distribution surrounding the Sn_2_ atom connects with the charge distribution between two phosphorus atoms. Another tin atom (Sn_1_) goes away from the middle of the P–P bond, and electron charge distribution surrounding the Sn_1_ atom is oriented to one of the sulfur atoms. Such difference in the hybridization of the electron orbitals for two types of tin atoms in the ferroelectric phase determines the disappearance of the layer-like anisotropy for the electron spatial charge distribution (Figure 8b).

## 6. Transformation of Electron Energy Spectra in the Transition from Paraelectric Phase into Ferroelectric Phase

According to experimental data [16–19], the chemical bonds and electron energy spectra of Sn_2_P_2_S_6_ crystal have noticeable changes in the ferroelectric phase transition. The calculated energy spectra demonstrates the changes in the energy gap and in the positions of all energy levels of the VB: the energies of electron state density peaks in the VB shift approximately by 0.5 eV (Figures 3 and 5). In the acentric phase the degeneration of electron energy levels disappears in some regions of the Brillouin zone, which determines the higher smearing of the energy distribution of electron density of states. It is important to mention that the lowering of the electron density of states near the top of the VB is also observed, together with a rise of the energy gap, in the transition into ferroelectric phase.

**Figure 9:**
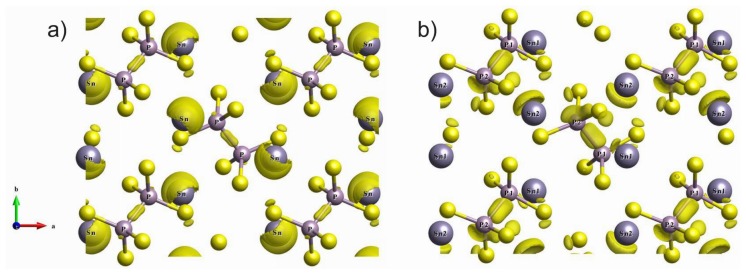
The electron density spatial distribution for the VI valence subband in the paraelectric (**a**) and the ferroelectric (**b**) phases of Sn_2_P_2_S_6_ crystal.

The changes of electron energy spectra obviously reflect an important role of electron–phonon interaction in the nature of the Sn_2_P_2_S_6_ crystal spontaneous polarization. Such interaction is illustrated by a transformation of the spatial electron density distribution in the change of the atom coordinates in the crystal structure. The squares of wave functions for electron orbitals, which are summed for the energy levels of valence subband VI in the paraelectric and the ferroelectric phases of Sn_2_P_2_S_6_ crystal, are shown in Figure 9. In the elementary cell of centrosymmetric structure, a similar distribution of electron density around four tin atoms is observed. This distribution reflects the stereoactivity of the lone electron pair 5*s*^2^ of cations Sn^2+^. Also, the spatial charge distribution around phosphorus atoms is similar. This is in agreement with the presence of the inversion center at the middle of P–P bond. In the acentric structure, the two pairs of tin atoms, with different distribution of the surrounding charge, appear. The nonequivalence of the electron density distribution near the phosphorus atoms is also seen. It should be mentioned that elevated electron density is located in the vicinity of neighboring tin atoms Sn_2_ and phosphorus atoms P_1_. Also, lowering of the surrounding charge is observed for the adjacent atoms Sn_1_ and P_2_. Acentricity of the P_2_S_6_ clusters is also reflected in deformation of the charge distribution along the P–P bonds.

Let us analyze peculiarities of the orbitals hybridization to study the tin 5*s*^2^ electrons stereoactivity and their contribution into lattice spontaneous polarization. In the crystal structure the tin atoms are placed in the polyhedrons that are created by eight sulfur atoms. At the beginning we will consider the mixing of tin orbitals with valence orbitals of surrounding sulfur atoms. Further, we will analyze the role of hybridization between tin atomic orbitals and P_2_S_6_ cluster molecular orbitals in short and long-range interactions in Sn_2_P_2_S_6_ crystal lattice, which induce ferroelectric phase transition.

## 7. Stereoactiviy of Lone 5s^2^ Electron Pair of Sn^2+^ Cations

Through the first-principles calculations of frozen phonons, it was found [8] that in Sn_2_P_2_S_6_ crystal the polar optic mode with symmetry *B_u_* in the paraelectric phase could be destabilized only on account of their nonlinear interaction with fully symmetrical *A_g_* optic mode. Generally, it is necessary to include the nonlinear interaction of *A_g_B_u_*^2^ type for all 13 *B_u_* and 15 *A_g_* normal vibrations of the lattice with symmetry *P*2_1_*/c* in the paraelectric phase. It could then explain the variation of the atomic coordinates at transition into the ferroelectric phase with symmetry *Pc*—two tin cations (that are related by the symmetry plane) have some shift relative to the anion sublattice, and another two tin cations are found to have a bigger shift (flipping) relative to their positions in the paraelectric phase. It was determined that effective potential in the normal *A_g_*–*B_u_* coordinates has three minima—the central one reflects the metastable paraelectric state, and the two side minima are related to two domains of the ferroelectric phase in uniaxial ferroelectrics.

The complicated potential relief for Sn_2_P_2_S_6_ crystal is obviously determined by relaxation of electron lone pair of tin cations. The electron pair in 5*s*^2^ configuration is stereoactive—*s* orbitals of cations are hybridized with *p* orbitals of sulfur. The antibonding energy level as a result of such hybridization is still occupied by electrons. For the energy gain, this level interacts with tin *p* orbitals—*sp*^2^ hybridization is realized for which the bonding level lowers their energy and antibonding level elevates the Fermi level. Such hybridization is proportional to the acentricity of the surrounding crystal field, and it induces deformation built by the dodecahedron of eight sulfur atoms surrounding the tin cation. Thereafter the metal cation shifts away from the dodecahedron center, and space distribution of the electron charge is clearly different from spherical. The stereoactivity of electron lone pair represents the second order Jahn–Teller effect (SOJT).

Already in the paraelectric phase, the tin cations have placed in general position of the elementary cell and their surrounding by electron density is definitely not spherical—almost rigid unaligned dipoles exist. Upon cooling the stereoactivity of electron lone pair growths and the orientation of cation shifts in the nearest elementary cells is correlated by dipole–dipole interaction. The spontaneous polarization appears with two contributions: “displacive” and “order/disorder”.

**Figure 10:**
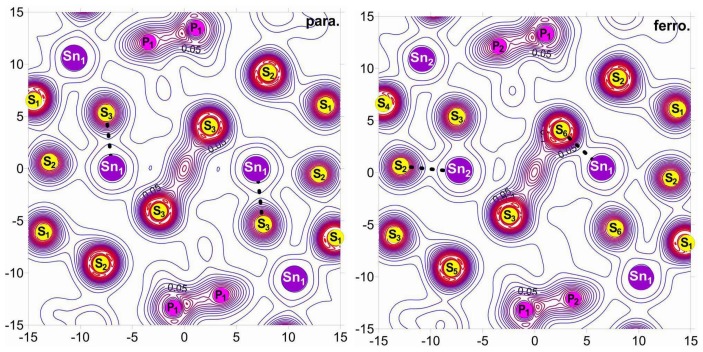
The transformation of the electron density space distribution in the vicinity of tin cations at the ferroelectric phase transition in Sn_2_P_2_S_6_ crystal. The strongest Sn–S bonds is shown by dashed lines.

The experimental data of XPS spectroscopy [19,30] about electron energy spectra near the top of VB in the paraelectric and the ferroelectric phases of Sn_2_P_2_S_6_ crystal confirm the lowering of the electron energy state density at transition into acentric structure. The calculations of energy spectra show big enough contribution of tin *s* orbitals into electron state density near the top of the VB and permit a possibility for tracing their change at the phase transition (Figure 5). The calculated space distribution of total electron density illustrates an evolution of electron lone pair at transition from the paraelectric phase into the ferroelectric one (Figure 10). The appearance of the nonequivalence of tin cations is observed: two of them shift in a direction of those sulfur atoms with which they have the biggest overlap of electron orbitals in the paraelectric phase; another two cations “flip” the biggest overlap of their electron orbitals in the direction of other sulfur atoms (Figure 10).

The high coordination of tin cations could be obviously described by taking into account their *d* orbitals. For symmetry requirements the hybridization of *sp*_2_*d*_5_ type satisfies and they could be related to the positioning of the tin cations inside of the eight caped polyhedron of sulfur anions. Indeed, the orbitals *s*, *p_x_*, *p_z_*, *d_xz_*, *d_xy_*, *d_zy_*, *d_z_*_^2^_, *d_z_*_^2^−_*_x_*_^2^_ transform on the irreducible presentations *A_g_*, *B_u_*, *B_u_*, *A_g_*, *B_g_*, *B_g_*, *A_g_*, *A_g_* (orientation of the monoclinic symmetry plane coincides with Cartesian plane XZ) that have a fully symmetrical combination.

**Figure 11:**
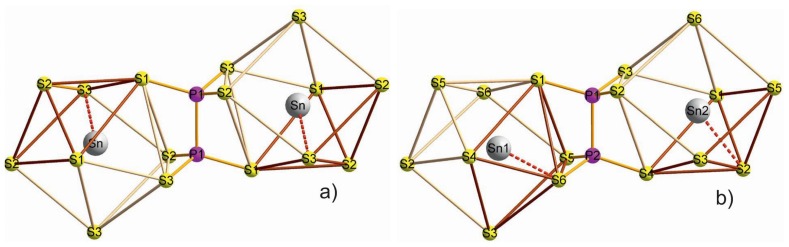
The transformation of sulfur atoms coordination polyhedrons around the tin cations at the ferroelectric phase transition in Sn_2_P_2_S_6_ crystal. The sulfur atoms that have stronger bonds with tin atoms are linked by dark lines. The strongest Sn–S bonds is shown by dashed lines.

In the elementary cell of the paraelectric phase, the four such dodecahedrons are equivalent—they pair-by-pair are related by a second order screw axis or by glade mirror plane (Figure 11a). In the ferroelectric phase as a result of the charge density redistribution discussed earlier, the inversion center and symmetry axis disappear. The two pairs of nonequivalent dodecahedrons appear. In one type of the dodecahedrons at the phase transition, the electron density switches between two nearest sulphur atoms which corresponds to the strongest Sn–S bond. In the other type of dodecahedrons at cooling from the centersymmetric phase to acentric one, the “flipping” of the electron density between almost oppositely oriented Sn–S bonds is observed (Figure 11b).

The sulfur atoms pyramids could be divided in the coordination polyhedrons (Figure 11). Localized at hybridized *sp*^2^-like orbitals of tin atoms, the electron density is oriented in direction opposite to the base of named pyramids. This is a direction to the three sulfur atoms with weaker Sn–S bonds.

Characterizing the chemical bonds changes at phase transition could be found by comparing the calculated data (Mulliken charges and overlap parameters for the electron orbitals of neighbor atoms) with experimental structure data, with Mössbauer, XPS and NMR spectroscopies data. For the paraelectric phase of Sn_2_P_2_S_6_ crystal, the following electron configurations were calculated: for four equivalence tin atoms Sn–5*s*^1:865^5*p*^1:153^5*d*^0:222^; for four equivalence phosphorus atoms P–3*s*^1:187^3*p*^2:508^3*d*^1:172^; for one of the three types of sulfur atoms S–3*s*^1:833^3*p*^4:231^3*d*^0:179^. It is evident that *d* orbitals are populated, which corroborates the explanation of high dodecahedral coordination of tin cations on the background of *sp*^2^*d*^5^ type hybridization. The highest population of *d* orbitals appears for phosphorus atoms. As was mentioned earlier, for the P_2_S_6_ clusters the bonds in PS_3_ structure pyramids are determined by *sp*^2^ hybridization of phosphorus atomic orbitals with their further *σ* hybridization with sulfur *p* orbitals. Thereafter one of two 3*s*^2^ electrons of phosphorus is involved into *sp*^2^ hybridization of atomic orbitals, and another electron is excited on the atomic *d* orbital. The named *sp*^2^ hybridization involves two *p* electrons of phosphorus, and a third *p* electron by *σ*(*p*–*p*) hybridization creates covalence P–P bond.

In the ferroelectric phase, the electron configurations for two types of tin atoms are the following: Sn_1_, 5*s*^1:851^5*p*^1:163^5*d*^0:224^; Sn_2_, 5*s*^1:850^5*p*^1:177^5*d*^0:226^. It is seen that at transition into the ferroelectric phase, the quantity of *s* electrons lowers by Δ*n_s_* = 0.014. Such calculations are in agreement with the observed lowering of an isomer shift for a spectral line of ^119^Sn Mössbauer effect at cooling from the paraelectric phase into the ferroelectric one [16].

The diminishing of *s* electron quantity in the ferroelectric phase for two types of tin atoms is compensated in a different step (level) by the growing of *p* orbitals occupancy. Hence the total charge of tin atoms changes from 3.240e in the paraelectric phase to 3.239e (Sn_1_) and 3.251e (Sn_2_) in the ferroelectric phase. The calculated rise of electron density in the vicinity of Sn_2_ atoms is in agreement with decreasing of resonance frequency in NMR spectrum for ^119^Sn from −781.3 ppm in the paraelectric phase to −782.5 ppm in the ferroelectric phase [18]. The increase of the resonance frequency till −754.5 ppm for Sn_1_ is obviously determined by occupancy redistribution from *s* orbitals to *p* orbitals at some lowering of total charge.

Determined by positions of XPS spectral lines [19], the energy of chemical binding for the 4*d* core orbitals in the ferroelectric phase differently increases for the two types of tin cations. This is obviously defined by the growing of 5*s*^2^ electrons’ lone pair stereoactivity and by these electrons’ redistribution on the *p* orbitals that are more distanced from tin cores.

The changes in Sn_2_P_2_S_6_ crystal structure at the spontaneous polarization appearance are characterized by the calculated values for the overlap parameters of electron orbitals of tin atoms and the nearest sulfur atoms. In the paraelectric phase such a parameter with the biggest value of about 0.054 has been found for the shortest bonds Sn–S in the dodecahedrons of sulfur atoms (Figure 11). It must be mentioned that in the nearest dodecahedrons, which are related by a second-order screw symmetry axis, such Sn–S bonds have opposite orientations of their projections onto plane (010), which contains the spontaneous polarization. In the ferroelectric phase, the nonequivalence of two pairs of cations Sn_1_ and Sn_2_ surround them and dodecahedrons of sulfur ions appear. Here the biggest overlap parameter for atomic orbitals (0.096) was found for the Sn_2_–S bond. This bond was strong already in the paraelectric phase and it is additionally enforced at the spontaneous polarization appearance—the atom Sn_2_ in the ferroelectric phase is shifted in the direction of sulfur atoms with the biggest content of chemical bonds covalency. For other coordination dodecahedra, the strongest chemical bond Sn_1_–S has the overlap parameter 0.087. Here at transition from the paraelectric phase into the ferroelectric one, the “flipping” of maxima in the space distribution of electron density occurs between bonds of the central metal atom and ligand atoms in the coordination polyhedron. As a result of such “flipping” in both types of the dodecahedron, the strongest bonds Sn_1_–S and Sn_2_–S have nearly oriented projections onto plane (010) (Figure 11b).

The changes in the electron density space distribution correlate with variations of the interatomic distances. For example, in the paraelectric phase the Sn–S bonds with the biggest overlap parameter (0.054) are strongest and they have the smallest length in the ferroelectric phase. For the dodecahedrons with tin atoms of Sn_2_ type, the overlap parameter for strongest bonds increases to 0.096 and their length decreases by 0.21 Å. Hence the occupancy of XZ plane oriented *p* orbitals raises from the calculations as follows: Δ*p_x_* = 0.048, Δ*p_y_* = −0.002, Δ*p_z_* = 0.022. For the dodecahedrons with tin atoms of Sn_1_ type at the electron charge density “flipping” on almost an oppositely oriented Sn–S bond, the overlap parameter, for the strongest bond in the paraelectric phase, lowers from 0.054 till 0.022 and the length of this bond growths by 0.26 Å. The strongest Sn_1_–S bond in the ferroelectric phase is characterized by the overlap parameter 0.087 and its length decreases by 0.35 Å. Here the changes for occupancies of tin *p* orbitals are: Δ*p_x_* = 0.025, Δ*p_y_* = 0.036, Δ*p_z_* = 0.010. We could see that cations of Sn_2_ type donate the biggest electronic contribution into the spontaneous polarization and this contribution has a “displacive” character. For the cations of Sn_1_ type, the electronic contribution is a little smaller and this one has an “ordering” character.

The calculated changes of Mulliken charges and the atomic orbitals’ overlap parameters coincide with the temperature dependence of the resonance frequencies in NMR spectra for ^31^P phosphorus [17,18]. In the paraelectric phase all phosphorus atoms are equivalent (calculated charge is 4.869e) and here only one NMR line with frequency 92.12 ppm is observed. At the spontaneous polarization appearance, the inversion center vanishes from what is associated with the growth of the phosphorus atoms’ nonequivalence in P_2_S_6_ clusters. For two types of phosphorus atoms the following electron configurations were calculated: P_1_–3*s*^1:1909^3*p*^2:499^3*d*^1:183^; P_2_–3*s*^1:182^3*p*^2:509^3*d*^1:193^. For atoms of P_1_ type with grown calculated charge (3.251e) in result of shielding effect, the NMR specter resonance frequency decreases to 89.2 ppm. This is in agreement with the closeness of P_1_ type atoms and Sn_2_ cations for which also a lowering of the NMR resonance frequency is observed [18] in the result of electron density growing in their vicinity at cooling into the ferroelectric phase. For atoms of P_2_ type in the ferroelectric phase, the resonance frequency rises till 93.7 ppm. In this case the weakening of the charge shielding effect is obviously determined by diminishing of the *s* orbitals occupancy. Moreover, for neighbor Sn_1_, cation of the quantity of *s* electrons also decreases, which induces growth of related resonance frequency in ^119^Sn NMR spectrum [18].

## 8. Discussion of Results

The calculated electron energy spectra, densities of electron states and their variation at transition from the paraelectric phase into the ferroelectric one coincide with the available structure data and results in the experimental investigation of the chemical bonds nature. On this ground the next generalized description of sources of the spontaneous polarization appearance in Sn_2_P_2_S_6_ crystal could be proposed.

The ferroelectric distortion is proportional to the difference between the Sn_1_ and Sn_2_ positions. For this distortion the short-range (mostly Sn–S) repulsions must be sufficiently small in order to allow to shift the equilibrium Sn positions from the center of the chalcogen dodecahedron. The effective charge of phosphorus has to be sufficiently small in order to allow the shift of the Sn cations in the direction of P–P bond. Such a requirement could be satisfied in the following way. At fully symmetrical *A_g_* lattice vibration, the important changes of electron charge distribution in the elementary cell occur—the charge is waded partially from anions P_2_S_6_ onto cations Sn. Hence the *p* orbitals of tin cations have to be occupied—the stereoactivity of valence electrons of these cations is realized by their partial hybridization with *p* orbitals of neighbor sulfur atoms. Such hybridization lowers the short-range repulsion between tin cations and the nearest sulfur atoms that govern their approaching. The charge transferring between tin and sulfur atoms manages some lowering of electrostatic interactions energy.

Generally, in the ground state (at 0 K) the metastable center-symmetric Sn_2_P_2_S_6_ structure is possible, for which both opposite tin atoms are slightly approached to the middle of the P–P bond. However, at low temperatures, the acentric structure is energetically more favorable. At the approach of two tin cations that are related by the inversion center at the middle of the P–P bond, their Coulomb repulsion increases (such repulsion between cations of tin and phosphorus obviously do not play an important role because positive effective charge of phosphorus is not big). Hence energetically more advantageous could be the approach of one tin cation to the center of the P_2_S_6_ cluster at the repulsion of the opposite tin cation. Indeed, in the center-symmetric structure both tin atoms are placed at the distance 3.633 Å, far from the middle of the P–P bond; in acentric structures such distances equal 3.463 Å for Sn_2_ atom and 3.857 Å for Sn_1_ atom. The space between considered tin atoms increases from 7.266 Å in the paraelectric phase and until 7.310 Å in the ferroelectric phase [14,20].

Removal of the Sn_1_ atom away from the P_2_S_6_ cluster decreases hybridization of its valence electron orbitals with molecular orbitals of cluster. Consequently, the electron charge in the cluster moves onto PS_3_ structural pyramid with P_1_ atom at their top that is the nearest to Sn_2_ atom. At repulsion of Sn_1_ atom from the cluster, an important change of the hybridization character occurs, which is accompanied by localization of valence electrons near the tin ion core and by growth of their kinetic energy. Such processes have an obvious activation character and they determine the presence of the energy barrier between central and side minima in the three-well potential.

In such a way, induced by the fully symmetrical *A_g_* vibration, the important changes of the charge gradient in the elementary cell determine variation of the electron configuration for the ions of crystal lattice. The reconstruction of electron configuration modifies the balance of interatomic interactions what induce instability of *B_u_* polar lattice vibration. Exactly by such manner, the mechanism of lattice modes *A_g_B_u_*^2^ nonlinear interaction could be presented, and this one governs the three-well potential presence for fluctuations of the order parameter of the ferroelectric phase transition in Sn_2_P_2_S_6_ crystal.

The value of the energy barrier in the three-well potential [8] equals near 0.015 eV, and the energy difference between central and side minima is near 0.01 eV. Such energetic characteristics are in agreement with our calculation of the electron energy spectra of Sn_2_P_2_S_6_ crystal in the paraelectric and the ferroelectric phases. Thus, for the ferroelectric phase, the full energy was found by 0.0078 eV smaller in comparison with calculated full energy for the paraelectric phase. It must be mentioned that at the crystal symmetry lowering the negative contribution of coulomb interactions into full energy decreases.

The appearance of spontaneous polarization in ferroelectric crystal is determined by variation of the chemical bonds covalency and by delicate balance between short-range repulsion forces, which determine the relief of local potential for the phase transition order parameter, and long-range displacement forces that define energy of intercell interaction.

The hybridization of tin and sulfur atomic orbitals which defines the appearance of “partially rigid” electric dipoles (pseudospins) as a result of tin cations valence electrons stereoactivity was analyzed earlier on. Such hybridization could be described as *sp*^2^ or *sp*^2^*d*^5^ combination of tin and sulfur atomic orbitals. This fact is clearly demonstrated by the presence of enough high density of Sn 5*s* states near the top of VB. In addition to that, the necessity of accounting for hybridization between tin atomic orbitals and P_2_S_6_ molecular orbitals has been found, which is argued for by presence of 5*s* and 5*p* states of tin even at the bottom of VB—in the energy range near −17.5 eV where the *s* states of phosphorus atoms are dominated.

The obtained pictures of the electron density space distribution show (Figure 7) the presence of –Sn–S–P–P–S–Sn– short-range bonds chains in the paraelectric phase. The occurrence of the short-range interactions in chains of – Sn–P–P –Sn– type have also been demonstrated. Such sequences of the short-range interactions together with long-distant coulomb interactions determine the mean-field, which induce correlation of the pseudo-spins at lowering of disordering influence of heat energy. At temperature down till 0 K, the chains of central symmetric structure groups–Sn–P_2_S_6_–Sn–, which are related to the pseudo-spins position in the central well of local potential, could obviously exist also. However, the correlated ordering of the structure motives (“dimers”) like –Sn –P_2_S_6_–, which responds to the pseudo-spins standing in one of the side well of local potential, is energetically more favorable.

It is important to remark about the rise of anisotropy of tin and phosphorus atoms surrounding in the ferroelectric phase (Figure 8) that support increasing their dynamic or Born effective charges. The dynamical transfers of charge are expected to be larger when such a hybridization involves *d* states, for which the interactions parameters with other orbitals are particularly sensitive to the interatomic distance [47]. Also, the amplitude of Born effective charges is not monitored by a particular interatomic distance but is dependent on the anisotropy of the Sn environment along the –Sn–S–P–P–S–Sn– chains. In the paraelectric phase, the S 3*p* electrons are obviously widely delocalized and dynamical transfer of charge can propagate along the – Sn –S–P–P–S–Sn– chains. In the ferroelectric phase, these chains behave as a sequence of “dimers”–Sn–S–P–P–S–. . . for which the electrons are less polarizable.

The anomalously large dynamical charges produce big LO-TO splitting for the ferroelectric soft phonon mode [48]. Moreover, this feature is associated with the existence of an anomalously large destabilizing dipole–dipole interaction, sufficient to compensate the stabilizing short-range forces and induce the ferroelectric instability. In materials where polar soft modes play a major role, the dynamical charge relate the electronic and structural properties [49]. However, for the Sn_2_P_2_S_6_ crystal the big LO-TO splitting for polar modes was not observed in phonon spectra [50,51]. Here, at 4.2 K such splitting is in the range of 2–7 cm^−1^. At heating to the temperature of phase transition in the ferroelectric phase, the LO-TO splitting for the lowest energy optic mode of *B_u_* symmetry (soft mode) reaches only the value of 10 cm^−1^.

The low frequency dielectric susceptibility temperature anomaly in Sn_2_P_2_S_6_ crystal is not described only by dielectric contribution of the polar lattice vibrations. On the data of dielectric spectroscopy [52] in the paraelectric phase, the dielectric contribution from polar lattice vibrations into static dielectric susceptibility reaches only near ten percent. Obviously, in the range of phonon frequencies, a significant destabilizing dipole-dipole interaction does not appear. The essential contribution into dielectric anomaly appears at a frequency lowering into submillimeter diapason—here the relaxational dispersion has been observed [53], which is obviously determined by nonlinear dynamic excitations.

The above attention was accented on the mixing between states of tin valence electrons and orbitals of phosphorus and sulfur atoms across all energy ranges of Sn_2_P_2_S_6_ crystal VB. In addition, the large enough density of phosphorus *s* and *p* states is presented near the top of VB also. Such phosphorus orbitals also create the conductivity band. The defined facts give evidence about strong mixing of diffusive *s* orbitals in the structure of Sn_2_P_2_S_6_ crystal. Obviously, the effective occupation of phosphorus *d* orbitals, which commonly are localized, also give evidence of their important role in the mechanism of electron–phonon interaction for this ferroelectrics.

For the P_2_S_6_ structure cluster, with symmetry *D*_3_*_d_* in free state, the 3*s* electron orbitals of phosphorus atoms, which are placed at bottom of the VB (near −15 eV) and their bonding *σ*(*p_z_* –*p_z_*) orbitals, which create P–P bonds and have an energy level near the top of VB (in the range of −1.5–0 eV), satisfy the transformation according to the *A_g_* irreducible presentation. In addition to these orbitals of free anion cluster, the orbitals with energies near −8.5 and −4 eV are also involved at the formation of P–P bond (Figure 2).

In the Sn_2_P_2_S_6_ structure the symmetry of P_2_S_6_ anion clusters is lowered, however, the possibility of effective mixing for the wave functions of *s* and *p* orbitals of phosphorus atoms, which have identical symmetry, is obvious. To the aforementioned series of four sets of P–P bonding orbitals, which create high electron density at the middle of this bond (Figure 6), the combination of tin atomic orbital related to the energy level 20 in VI subband (in the range from −8 to −6.5 eV) is added. Obviously, as a result of such hybridization, the similar changes at the top and bottom of the VB are observed at the appearance of spontaneous polarization in the crystal (Figure 5).

Generally, the high effective charge and large polarizability of P_2_S_6_ anionic clusters together with stereoactivity of tin electron lone pairs determine large electronic contributions from all atoms into spontaneous polarization of Sn_2_P_2_S_6_ crystal. Such a situation is in agreement with earlier findings [8] involving of all 13 *B_u_* modes and 15 *A_g_* modes to dynamic instability of investigated ferroelectrics. However, the obviously important role also belongs to the nonlinear interaction with participation of *A_u_* and *B_g_* nonsymmetric modes. For the point group 2*/m* in addition to the *A_g_B_u_*^2^ fully symmetric combination, the invariants of *A_u_B_g_B_u_* type are also present. A significant role of such nonlinear mixing of lattice vibrations is reflected in strong internal deformations of P_2_S_6_ clusters at the phase transition. In the crystal electron structure, such invariants obviously replicate the hybridization of molecular orbitals of P_2_S_6_ clusters with participation of atomic *d* orbitals. Such hybridization is clearly illustrated by the spatial distribution of electron density for the energy level number 20, which is aligned from tin atoms to the middle of P–P bond (Figure 7).

The energy decreasing for the *s* orbitals of phosphorus and sulfur at the VB bottom (their contribution is dominated in the lowest subbands, from I to V), and also lowering of energy for tin *s* orbitals, with their contribution across whole range of the VB (Figure 5), are obviously essential for energetic motivation of the transition into the ferroelectric phase. Also some lowering of the *p* and *d* orbitals energy occurs.

The electronic structure XPS measurements for Sn_2_P_2_S_6_ crystals [19,30] revealed the chemical shifts of Sn and P electronic core states to a higher binding energy and of S states to a lower binding energy at the crystal lattice formation. This shift suggests a charge transfer from Sn and P to S atoms. The binding energies and chemical shifts strongly change at the phase transition. In the ferroelectric phase, the chemical shifts of Sn and P atoms are higher while for S atoms they are smaller. So, for all atoms of crystal structure at transition into the ferroelectric phase the binding energy for core orbitals increases. These data give evidence about localization of electron charge in space between atoms, or about enhancement of chemical bonds covalency. Such variation of the core orbitals energy agrees with what was experimentally observed about transformation of the VB structure (Figures 3 and 5) and that they support an energetic stability of the ferroelectric phase.

Since covalency increases, there might be a possibility for the drastic collapse of the sulfur ionic size (which is related to charge transfer). For the S^2−^ ion the ionic radius is 1.84 Å, and the covalent radius *≈* 1.02 Å [54]. If the size of sulfurs were small compared to the allowed space then the sulfur atoms would be weakly bound in the lattice. In this case, an imbalance between the decreased (due to small S radius) repulsive forces and the polarization forces tend to displace the ion from its position and also support structure rearrangement.

On the whole, for Sn_2_P_2_S_6_ crystal at the transition from the paraelectric phase to the ferroelectric, one complex evolution of electron and phonon spectra occurs, which could be presented as a sequence of five steps. Evidently a *first* factor assists the change of the electron density charge distribution in the elementary cell by fully symmetric breathing modes *A_g_*. Such redistribution of the electron density prompts the stereochemical activity of tin cations electron lone pair and produces the covalence bonds of tin atoms with sulfur atoms (hybridization of *sp*^2^*d*^5^ type), and also with phosphorus atoms, that could be considered as the *second* part.

A *third* stage could be considered the weakening of the short-range repulsion between cations of tin and phosphorus, as a result of their charges lowering, and at significant coulomb repulsion of the nearest (related by the inversion center) tin cations. The second and the third factors represent the nonlinear interaction of *A_g_B_u_*^2^ type; they govern an anisotropy of polar shifting of atoms in the elementary cell and define appearance of the dipole structure motives (–Sn–P_2_S_6_–), which are related to the polar normal coordinates of *B_u_* symmetry. The *fourth* important factor is the dipole–dipole interaction which correlates orientation of local dipoles (pseudospins) and defines appearance of the spontaneous polarization in the crystal structure. Finally, the *fifth* circumstance illustrates that all low symmetry modes participate as a result of permitted nonlinear *A_u_B_g_B_u_* relation in the structure transformation. Such combination of structure deformations mirrors participation of phosphorus and sulfur *d* orbitals in the covalent bonds of Sn_2_P_2_S_6_ crystal.

It is interesting to compare the peculiarities of chemical bonds in Sn_2_P_2_S_6_ sulfide and Sn_2_P_2_Se_6_ se- lenide compounds, and also in the lead in contained Pb_2_P_2_S_6_ crystal. At first, we will consider the binary compounds MX, where M is the metal Ge, Sn, Pb and X is the chalcogen O, S, Se, Te. The stereoactivity of electron lone pair for metal atoms is determined by *sp*^2^ hybridization of their *s* and *p* orbitals with *p* orbitals of chalcogen atoms. Such hybridization is determined by the positions of the energy levels of electron states and by width of the related energy bands in the crystal structure [48,55]. The smallest energy difference is present between positions of energy levels of germanium *s* orbitals and oxygen *p* orbitals. Consequently for the compound GeO, the largest stereoactivity of 4*s*^2^ electron orbitals of Ge is observed [55]. At transition from Ge to Sn and than to Pb, the energy of chemical binding for the *s* orbitals increases. Thus with transition from O to S, and further to Se and Te, the energy of chemical binding for their *p* orbitals decreases. It is expected that the hybridization of Ge 4*s* orbitals and O 2*p* orbitals is the strongest, and hybridization for Pb 6*s* orbitals and Te 5*p* orbitals is the most weak. However the hybridization is also influenced by width of related energy bands in the crystal structure. The increase of width for the *s* and *p* electron states energy bands and their overlap could partially compensate increase of the energy distance between related energy levels, which produce some level of the stereoactivity and covalency of M–X bonds.

Thus, at transition from Sn_2_P_2_S_6_ to Pb_2_P_2_S_6_ the binding energy for Pb 6*s* level increases, which weakens the stereoactivity of the 6*s*^2^ electron lone pair in the dodecahedron of sulfur atoms. Obviously, the observed [56] ionicity for the Pb–S bonds is higher in comparison with Sn–S bonds ionicity. The melting temperature and the energy gap both rise at substitution Sn by Pb. The paraelectric phase in Pb_2_P_2_S_6_ is stable at cooling till 4.2 K [7].

The largest stereoactivity of Ge 4*s*^2^ electron lone pair surrounding sulfur atoms gives a natural explanation of absence of Ge_2_P_2_S_6_ crystal structure. The Ge atoms could not be placed in positions with high coordination of sulfur atoms. Obviously introducing Sn_2_P_2_S_6_ crystal impurity of germanium in the charge state Ge^2+^ strongly elevates the temperature of ferroelectric phase transition that was observed by dielectric investigations [24]. Certainly the impurity in charge state Ge^4+^ will not be stereoactive and will not support a rising temperature interval for the ferroelectric phase existence.

According to these described tendencies, at transition from Sn_2_P_2_S_6_ to Sn_2_P_2_Se_6_ the stereoactivity of Sn 5*s*^2^ electron lone pair in the dodecahedron of selenium will be smaller than in the case of sulfide compound. However, for the ternary compounds the ion-covalence bonds Sn–S and Sn–Se are modified depending on peculiarities of P–S and P–Se bonds in P_2_S(Se)_6_ anion clusters. On the Mössbauer spectroscopy data [56] at substitution of sulfur by selenium, the isomer shift for ^119^Sn nucleus decreases what directly show on higher covalency of Sn–Se bonds. The NMR spectroscopy for ^119^Sn [18,57] shows an increase of the resonance frequency from −781.3 ppm in Sn_2_P_2_S_6_ to −608 ppm in Sn_2_P_2_Se_6_. The NMR spectral line for ^31^P decreases its frequency from 92.12 ppm in Sn_2_P_2_S_6_[18] to 28.7 ppm in Sn_2_P_2_Se_6_[57]. These data provide evidence about the lowering of the electron density in vicinity of tin nucleus and about their rise at phosphorus nucleus at transition from sulfide to selenide compound. The mentioned tendency could be explained by smaller electronegativity of selenium. Obviously the bonds P–Se are less polar, which improve higher electron charges of phosphorus atoms in anionic clusters. Such a situation probably also supports more effective hybridization between cluster molecular orbitals and tin atomic orbitals (mostly the bonding hybridization of tin orbitals with molecular orbitals that are localized in the middle of P–P bond) and increases the stereoactivity of tin lone pair of electrons in Sn_2_P_2_Se_6_ crystal.

The growth of covalency and weakening of electrostatic interactions determine the lowering of the melting temperature for Sn_2_P_2_Se_6_ crystal in comparison with sulfide analog, and defines the decrease of the energy gap while governing a lower temperature of the ferroelectric phase transition [7].

## 9. Conclusions

The appearance of the spontaneous polarization in the Sn_2_P_2_S_6_ compound is accompanied by the significant changes of an electronic structure that are observed in all subbands of this crystal VB. At the transition from the paraelectric phase to the ferroelectric one, the significant changes also occur for the phonon spectra in the whole frequency range—for both external and internal vibrations of the crystal lattice. The complicate evolution of the energy spectra could be represented by the following contributions. The fully symmetrical (“breathing”) *A_g_* modes change the space distribution of the electron density in the elementary cell. This one initiates the stereochemical activity of the tin cations electron lone pair and support creation of their covalence chemical bonds with sulfur atoms (the hybridization of *sp*_2_*d*_5_ type), and with phosphorus atoms also. Thus as a result of the ionic charges lowering, the short-range repulsion between tin and phosphorus cations decreases, however, the coulomb repulsion between tin cations still remains strong enough. These factors reflect the nonlinear interaction of the *A_g_B_u_*^2^ type, they determine the anisotropy of polar deformations in the elementary cell and induce appearance of the dipole structure motives (–Sn–(P_2_S_6_)–), which are related to the polar normal coordinates of *B_u_* symmetry. The dipole–dipole interaction correlates orientation of the local dipoles (pseudospins) and governs the spontaneous polarization of the crystal structure. In the structure rearrangement, all low symmetry modes take part; the possibility of the nonlinear linking of *A_u_B_g_B_u_* type is obvious. This interaction correlates with the involvement of tin and phosphorus *d* orbitals into creation of the covalent bonds in Sn_2_P_2_S_6_ crystal lattice. A small difference of the paraelectric and ferroelectric phase’s energies and activation redistribution of the electron charge at the spontaneous polarization appearance determines the presence of the three-well local potential in the Sn_2_P_2_S_6_ ferroelectrics.

## Figures and Tables

**Table 1:** Characters of irreducible representations of the little groups of Γ(0*,* 0*,* 0) and $A(-\frac{1}{2},0,\frac{1}{2})$ points. Space group *P*2_1_*/n*.

	*E*	*C̃*_2_	*I*	*σ̃_h_*
Γ_1_ (*A*_1_)	1	1	1	1
Γ_2_ (*A*_2_)	1	1	−1	−1
Γ_3_ (*A*_3_)	1	−1	1	−1
Γ_4_ (*A*_4_)	1	−1	−1	1

**Table 2:** Characters of irreducible representations of the little groups of $D(0,\frac{1}{2},\frac{1}{2})$ and $C(\frac{1}{2},\frac{1}{2},0)$ points. Space group *P*2_1_*/n*.

	*E*	*C̃*_2_	*I*	*σ̃_h_*
{*C*_1_ ⊕ *C*_3_} ({*D*_1_ ⊕ *D*_3_})	2	0	2	0
{*C*_2_ ⊕ *C*_4_} ({*D*_2_ ⊕ *D*_4_})	2	0	−2	0

**Table 3:** Characters of irreducible representations of the little groups of $B(0,0,\frac{1}{2}),Y(\frac{1}{2},0,0),Z(0,\frac{1}{2},0)$ and $E(-\frac{1}{2},\frac{1}{2},\frac{1}{2})$ points. Space group *P*2_1_*/n*.

	*E*	*C̃*_2_	*I*	*σ̃_h_*
*B*_1_ (*Y*_1_,*Z*_1_,*E*_1_)	2	0	0	0

**Table 4:** Characters of irreducible representations of the little groups of Γ(0*,* 0*,* 0), $A(-\frac{1}{2},0,\frac{1}{2}),Z(0,\frac{1}{2},0)$ and $E(-\frac{1}{2},\frac{1}{2},\frac{1}{2})$ points. Space group *Pn*.

	*E*	*σ̃_h_*
Γ_1_ (*A*_1_, *Z*_1_, *E*_1_)	1	1
Γ_2_ (*A*_2_, *Z*_2_, *E*_2_)	1	−1

**Table 5:** Characters of irreducible representations of the little groups of $D(0,\frac{1}{2},\frac{1}{2}),C(\frac{1}{2},\frac{1}{2},0),B(0,0,\frac{1}{2})$ and $Y(\frac{1}{2},0,0)$ points. Space group *Pn*.

	*E*	*σ̃_h_*
{*D*_1_ ⊕ *D*_2_} ({*C*_1_ ⊕ *C*_2_}, {*B*_1_ ⊕ *B*_2_}, {*Y*_1_ ⊕ *Y*_2_})	2	0
